# Comparing New Smartphone-Connected Handheld Ultrasound Device vs. Traditional Ultrasound in Vitreo-Retinal Disease Diagnosis

**DOI:** 10.3390/diagnostics14171961

**Published:** 2024-09-05

**Authors:** Giovanni Rubegni, Marco Zeppieri, Andrea Russo, Niccolò Castellino, Mario Fruschelli, Theodora Hadjistilianou, Linda Tognetti, Matteo Orione, Luca Lanzafame, Caterina Gagliano, Alessandra Cartocci, Gian Marco Tosi, Teresio Avitabile

**Affiliations:** 1 Ophthalmology Unit, Department of Medicine, Surgery and Neurosciences, University of Siena, 53100 Siena, Italy; giovannirubegni@gmail.com (G.R.);; 2 Department of Ophthalmology, University Hospital of Udine, 33100 Udine, Italy; 3 Department of Ophthalmology, University of Catania, 95123 Catania, Italy; 4 Dermatology Unit, Department of Medical, Surgical and Neurological Sciences, University of Siena, 53100 Siena, Italy; 5 Eye Clinic, Polytechnic University of Marche, 60125 Ancona, Italy; 6 Department of Medicine and Surgery, University of Enna “Kore”, 94100 Enna, Italy

**Keywords:** vitreo-retinal diseases, POCUS, telemedicine

## Abstract

(1) Background: Ocular emergencies account for 1.5–3% of emergency department (ED) visits and require urgent diagnosis to prevent serious complications. Ultrasonography is a crucial, non-invasive diagnostic tool for these conditions but traditionally lacks portability and integration with modern electronic smart devices. The purpose of this study was to assess the accuracy and performance of a new handheld ultrasound device in comparison to a conventional cart-based sonographic machine in patients attending to the ED for vitreo-retinal diseases. (2) Methods: three specialists in ophthalmology, with at least 4-year experience in vitreo-retinal diseases and eye ultrasound, evaluated images of 50 eyes with both portable and traditional ultrasound probes. Each specialist made the diagnosis based on the images captured with both probes and then rated their overall image quality and confidence of diagnosis with a five-point Likert scale. The concordance of diagnosis between the two probes was evaluated. (3) Results: The sample comprised 42 patients. Twenty (40%) healthy eyes and thirty eyes with the following vitreo-retinal interface conditions were examined: 12 retinal detachment (24%), 8 vitreous hemorrhage (16%), and 10 posterior vitreous detachment (20%). The overall accuracy of the two devices appeared to be comparable (70.7% vs. 69.3%). The Butterfly iQ+ probe showed similar sensitivity in retinal detachment diagnosis (91.7% vs. 94.4% of the Accutome B-scan Pro), while it showed poor performance in diagnosing posterior vitreous detachment (sensitivity = 27.2%); (4) Conclusions: The Butterfly iQ+ device demonstrated high sensitivity in the diagnosis of retinal detachment. Significant adjustments are still needed to improve the resolution of the vitreous body.

## 1. Introduction

Ocular emergencies account for 1.5–3% of all emergency department (ED) visits [1]. The acute symptoms most often reported by the patients are sudden vision loss, flashing lights (photopsia), and floaters (myodesopsia). The most common conditions that can cause these symptoms include posterior vitreous detachment (PVD), retinal tear (RT), rhegmatogenous retinal detachment (RD), as well as other ocular diseases such as vitreous hemorrhage (VH). On these bases, this symptomatology requires both prompt and accurate assessment for an underlying treatable pathology. Timely treatment interventions, such as laser or surgery for RT or RD, are necessary to reduce the risk of progressive visual impairment and blindness associated with these disorders [2,3]. Clinically, it is critical to distinguish between these three disorders because. In contrast, patients with RD may require emergency examination by an ophthalmologist, and those with VH and PVD can frequently be discharged with attentive outpatient follow-up [4].

Ophthalmologists have used eye ultrasonography for decades as an essential technique for evaluating vitreo-retinal diseases and other conditions for its capability to obtain noninvasively reliable information in a wide range of settings [5]. According to recent studies, point-of-care ultrasound (POCUS) devices in the ED setting can detect RDs with an average sensibility and specificity comparable to traditional eye ultrasound systems [6,7,8]. However, both conventional ophthalmic B-scan and eye POCUS lack portability and integration with modern smart devices [9]. Recent technological advances have made it possible to create pocket-sized ultrasound machines (HHUS) that connect directly to modern smart devices, overcoming many previous limitations [10]. In a variety of applications, such as bedside procedures like thoracentesis and epidural analgesia, as well as diagnostic evaluation of reproductive organs, cardiac function, lung function and anatomy, abdominal pathologies (renal disorders, abdominal aortic aneurysm), and musculoskeletal systems, handheld wireless ultrasound devices have shown comparable accuracy to cart-based ultrasound machines [10,11,12,13,14,15,16,17]. Few studies have investigated the capabilities of these devices in the ophthalmic field, primarily assessing ultrasound image quality [18,19].

However, to date, there is scarce data on the performance of these new portable ultrasound devices compared to traditional eye ultrasound machines in ophthalmology practice. Therefore, the purpose of this study is to evaluate and compare diagnostic performances and image characteristics of B-scans obtained by a novel handheld multipurpose ultrasound machine (the Butterfly iQ+) and our current mid-range ultrasound system (Accutome B-scan Pro) in vitreo-retinal disease evaluation in an emergency setting.

## 2. Materials and Methods

### 2.1. Setting

This observational study was carried out in a tertiary care center, “Azienda Ospedaliera Universitaria Policlinico “G.Rodolico”, from November 2022 to November 2023. The study was conducted as stated by the Declaration of Helsinki. Images were acquired during the routine clinical activity of the outpatient vitreo-retinal surgery clinic and of the emergency room consultation.

### 2.2. Subject Selection

A total of 42 patients were enrolled, and 250 B-scans of 50 eyes were obtained by each of the multipurpose handheld US Butterfly IQ+ (Butterfly Inc., Burlington, USA) and the conventional ophthalmic US Accutome B-scan Pro (Accutome Inc., Malvern, USA). Patients above 18 years of age admitted to the ophthalmology ED were included in the study. Patients were excluded if they had undergone previous retinal surgery and in cases of recent eye trauma or corneal thinning/perforation.

### 2.3. Ultrasound Probes

Accutome B-scan PRO (Accutome Inc., Malvern, PA, USA) uses conventional ultrasonic emission technology with piezoelectric materials. The mechanically scanned focused single-element probe (10 MHz) was used. The handheld Butterfly iQ+ (Butterfly Network Inc., Burlington, MA, USA) relies on capacitive micro-machined ultrasound transducers (CMUTs), allowing for changes in MHz as a preset function (a single probe can scan at different MHz).

### 2.4. Image Acquisition

**Figure 1:**
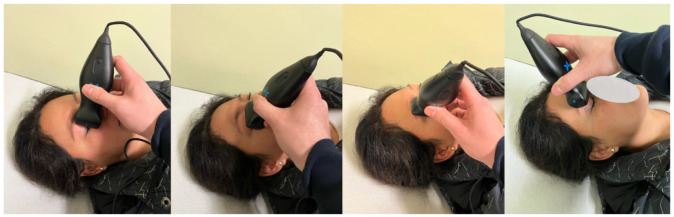
Butterfly iQ+ probe positioning during image acquisition.

The images captured by both devices were acquired by the same operator experienced in ultrasonographic diagnostics of vitreo-retinal interface diseases (NC). The scan with the handheld Butterfly iQ+ was acquired by selecting an ophthalmic preset from the drop-down menu; this preset uses an ultrasound frequency of 10 MHz. The sonographer was instructed to adjust the gain, depth, and frequency of each probe to optimize the best picture on each machine. The examination with both probes was conducted in the same manner. Transverse scans of the eye were obtained at the following gaze positions: posterior pole, temporal, nasal, inferior, and superior quadrants (Figure 1).

The obtained images were cropped, deidentified, and masked, leaving only the gray-scale images of the scans.

### 2.5. Image Evaluation

**Figure 2:**
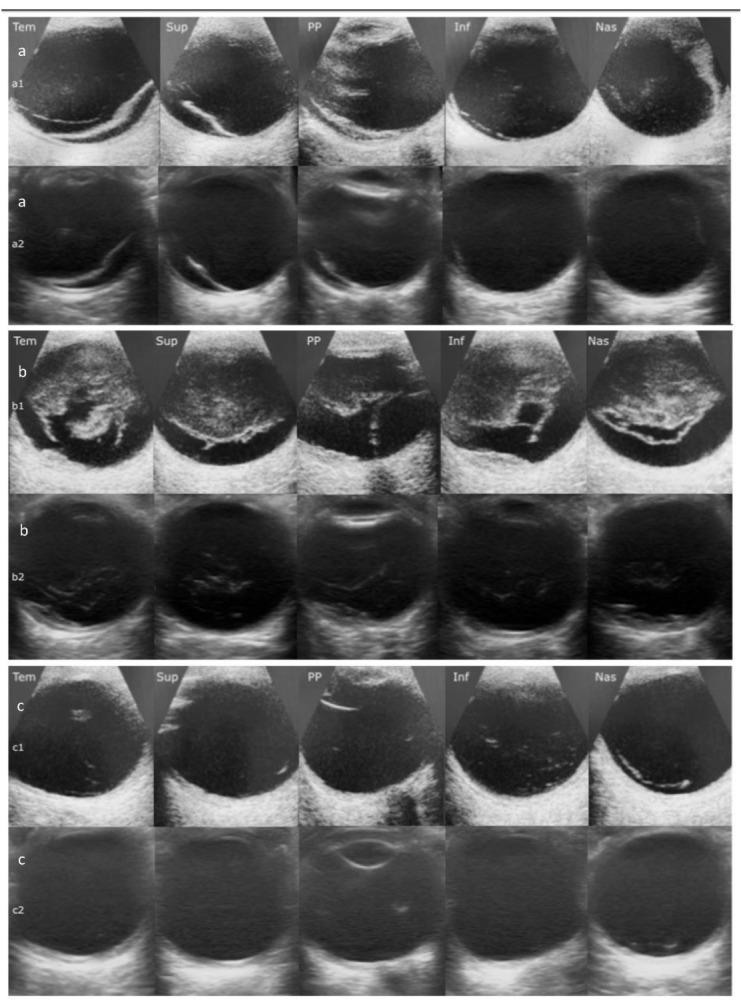
(**a**) Retinal detachment as seen by the Accutome (**a_1_**) and iQ+ (**a_2_**) probes in the five transverse projections (posterior pole, superior, inferior, temporal, and nasal); (**b**) Vitreous hemorrhage as seen by the Accutome (**b_1_**) and iQ+ (**b_2_**) probes in the five transverse projections; (**c**) Posterior vitreous detachment as seen by the Accutome (**c_1_**) and iQ+ (**c_2_**) probes in the five transverse projections.

Three experienced eye sonologists from different university hospitals (Azienda Ospedaliero Universitaria Policlinico G. Rodolico—San Marco, Azienda Ospedaliero-Universitaria Senese, Azienda Ospedaliero Universitaria delle Marche) with more than four years of experience in eye ultrasonography and vitreo-retinal interface disorders (MO, LL, and MF) evaluated the images. Each reviewer evaluated 100 images (i.e., 50 images per device). Possible diagnoses were healthy, retinal detachment, vitreous hemorrhage, and posterior vitreous detachment. A ground truth diagnosis was made by a vitreo-retinal disease specialist with more than 15 years of experience (AR) based on the patient’s medical history and clinical examination (fundus examination, OCT, eye ultrasound). Retinal detachment was defined as a hyperreflective taut linear opacity within the vitreous chamber. Vitreous hemorrhage was described as wavy linear or curved strands in the vitreous cavity accompanied by variable degrees of vitreal opacity. Posterior vitreous detachment is described as a fine swaying line that appears anteriorly to the retina where the vitreous seems detached [20]. (Figure 2a–c).

For each evaluation, they also graded diagnostic confidence and image quality (overall assessment encompassing contrast of solid and fluid-filled structures and the absence of noise). Each characteristic was rated using a five-point Likert scale (0 = poor to 5 = excellent).

### 2.6. Statistical Analysis

Descriptive statistics were carried out. Qualitative variables are summarized with absolute frequencies and percentages. Instead, quantitative variables with mean ± standard deviation. Global accuracy, sensitivity, and specificity for each diagnosis with their 95% confidence interval (95% CI) were estimated. The sensitivity and specificity of the two devices were compared with the proportion test. The Fleiss Kappa for more than two raters was estimated to evaluate the concordance between raters for each device. The Cohen Kappa for two raters was estimated to assess the agreement between the two devices.

A *p*-value < 0.05 was considered statistically significant. All the analyses were carried out with R version 4.3.2.

## 3. Results

### 3.1. Study Sample

The sample comprised 42 patients. A total of 18 (42.9%) are female, and the mean age is 57.5 ± 11.4. Twenty (40%) healthy eyes and thirty eyes with the following vitreo-retinal interface conditions were examined: 12 (24%) RD, 8 (16%) VH, and 10 (20%) PVD.

### 3.2. Diagnostic Accuracy

Retinal detachment diagnosis was not correctly classified in two cases with the Accutome B-scan Pro and three cases with Butterfly iQ+. The sensitivity of RD diagnosis was 94.4% with Accutome B-scan Pro and 91.7% with Butterfly iQ+. The specificities were 91.2 and 95.6, respectively (Table 1).

**Figure 3:**
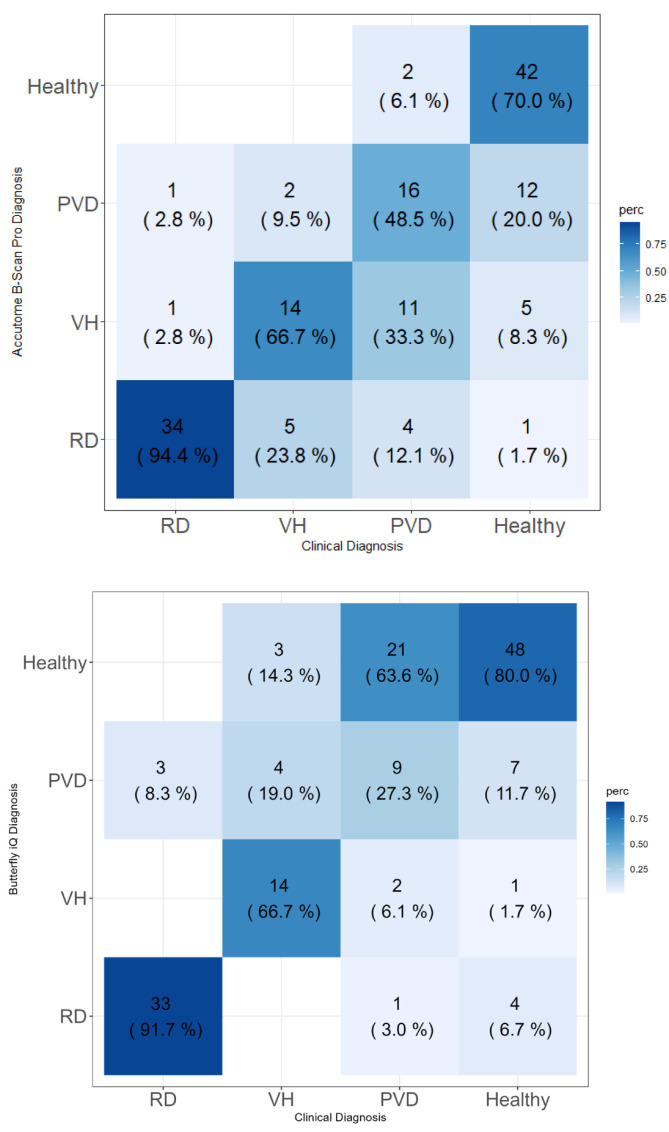
Confusion matrix of the comparison between the clinical diagnosis and the direct diagnosis made with Accutome B-scan Pro and Butterfly iQ+.

No statistical difference was observed in terms of sensitivity and specificity. The sensitivity of VH diagnosis was similar for both devices (66.7%); however, Butterfly iQ+ had significantly higher specificity. The Butterfly iQ+ probe showed lower sensitivity than the Accutome probe in distinguishing normal eyes from any pathology (73.3% vs. 97.8%). However, Butterfly iQ+ had 10% higher specificity in identifying healthy eyes than the Accutome (Figure 3).

The overall accuracy of the two devices appeared to be comparable (70.7% vs. 69.3%).

### 3.3. Agreement on Diagnosis

The agreement among the three evaluators on the diagnosis was 0.569 for the traditional probe and 0.552 for the Butterfly iQ+ (Table 2).

Notably, we observed a higher agreement falling within the range of good for the diagnosis of RD. The observed values were comparable between the two probes: 0.775 for Accutome B-scan Pro and 0.789 for Butterfly iQ+. The diagnosis showing a lack of agreement or poor agreement was PVD.

However, regarding RD, which was the condition in which both probes proved to be most accurate, the agreement was moderate. 

Globally, the two probes showed poor agreement, especially regarding PVD, which appeared to be not significant (Table 2).

### 3.4. Rating on Image Quality

Table 3 reports the distribution of ratings by the three raters on image quality stratified by diagnosis and probe type.

The image quality captured with the Butterfly iQ+ probe was found to be at least moderate in 70% of cases. In the case of RD diagnosis, images were classified as high/very high in 69.4% of cases compared to 77.8% with the standard probe. However, the latter achieved an excellent rating in 30% of assessments. For the PVD diagnosis, the standard probe proved superior in image quality. Regarding healthy cases, however, the Butterfly iQ+ was rated as high/very high in +15% of cases.

There was significant confidence in diagnosis (Table 4), with raters reporting high/very high confidence in 85% of cases with the Accutome probe, while it was only 50% for the Butterfly iQ+ probe.

## 4. Discussion

In this study, we compared the diagnostic accuracy and image characteristics of a new handheld general-purpose ultrasound probe versus a traditional ultrasound device in an ophthalmological emergency setting. The opportunity to have a portable and inexpensive instrument available for rapid diagnosis of posterior segment disorder could offer relevant advantages, especially in the field of teleophthalmology.

Image quality assessment on scans captured with the Butterfly iQ+ probe was found to be at least good in 70% of cases, while the traditional probe was rated at least good in 62.2%. The Accutome probe demonstrated slightly higher ratings in pathological cases (66.7% vs. 58.5%) and lower ratings in healthy patients (48.4% vs. 63.4%). The most significant difference was found in PVD cases in which Accutome probe images were rated as at least good in 60.6% versus 39.4% for the iQ+ probe. Image quality evaluations seem to align with the results on the diagnostic performance of the two probes, which showed comparable diagnostic accuracy for all diagnoses except in the PVD case.

In our analysis, the iQ+ probe showed good general accuracy (69.3%), with particularly high sensitivity in detecting retinal detachment (sensitivity = 91.7%). A high concordance value (0.698) with the traditional probe was observed, too. In vitreous hemorrhage, the sensitivity between the two probes was exactly the same at 66.7%, although iQ+ specificity significantly differed from the traditional probe (86.8% vs. 97.7%). In contrast, the result of posterior vitreous detachment evaluation was profoundly different. The butterfly probe showed poor sensitivity in diagnosing this condition (27.3%), while the traditional probe was superior, although with unsatisfying diagnostic accuracy (sensitivity = 48.5%). No concordance between examiners was revealed in PVD cases evaluated by the iQ+ probe. Finally, the concordance with the traditional probe in this condition appeared close to 0 (0.051). Numerous studies have examined the performance of bedside ultrasonography (POCUS) in the diagnosis of retinal detachment [1,21,22]. According to a recent review [6] analyzing 844 patients from 11 studies, bedside ultrasonography showed a sensitivity of 92.0% and specificity of 91.4%. These results align with those obtained by the iQ+ probe. Similar results for RD diagnosis were recently reported by Lahhman S et al. [23]. Here, the POCUS probe demonstrated a sensitivity of 96.9% and specificity of 88.1% for the diagnosis of retinal detachment, 81.9% sensitivity and 82.3% specificity for vitreous hemorrhage, and 42.5% sensitivity and 96.0% specificity for vitreous detachment. In this case, the results on hemovitreous, especially in posterior vitreous detachment, are very similar to those obtained in our study by the conventional probe, confirming a lower diagnostic capability of the iQ+ probe in vitreous evaluation.

This limitation could be due to the high signal-to-noise ratio of the iQ+ probe under 20 mm of distance and to the high generalized contrast-to-noise ratio for targets with low echogenicity [24]. This technical restriction could be mitigated by changing the settings in the probe software, but, to date, the ophthalmic setting is the only one approved by the FDA (and EU MDR) for use on the eye. Improvement could still occur with a software update, considering the technical requirements for diagnosing vitreoretinal diseases. This point is crucial to make the probe self-sufficient in a screening/emergency setting. Another limitation demonstrated by the iQ+ probe was its size (163 × 56 mm), which often made it difficult to maneuver, especially in patients with sunken eyes. This difficulty was never such that the examination was not performable. Considering both vitreous disorders, VH and PVD, it is evident that the two probes showed disappointing accuracy results. This may be due to two reasons. First, when evaluating these images, the ophthalmologists were unaware of the patient’s clinical data, such as visus oculi, anterior segment, and fundus oculi examination. This factor is crucial since, in clinical practice, the specialist during an ultrasound examination has elements that enable him to interpret the images correctly. Not providing such data to examiners was an intentional choice because, in an emergency care setting, such data are not available to the emergency medicine physician. Second, the evaluation was done on static images, thus not taking full advantage of the ultrasound probe’s ability to dynamically assess the movements of the vitreal contents and underlying retina.

A recent article investigated the performance of this probe in diagnosing retinal detachment alone. In this study, the probe obtained sensitivity and specificity results of 95% and 100%, which agreed with those obtained in our work [25].

The results of the diagnostic capabilities of Butterfly iQ+ are particularly noteworthy, considering its extreme portability (dimension H × W × D: 163 × 56 × 35 mm; weight: 309 g) and affordability (currently 2.300 Euros), when compared to conventional ultrasound devices (dedicated eye ultrasound probe and POCUS). Additionally, Butterfly iQ+ has integrated software that enables the sharing of ultrasound pictures/videos directly over the internet and makes it easier for US professionals to provide remote assistance in the analysis of sonographic exams. This method may eventually allow ophthalmologists to supervise and assess ultrasonography tests carried out by medical professionals or patients from a distance using telemedicine. For example, this device could be used by emergency medicine physicians to evaluate patients who report floaters, photopsia, or scotomata in the ED, sending exam videos directly to the ophthalmologist.

This study had some limitations. First, a low number of patients was evaluated. However, the number of cases is consistent with previous, similar studies [26,27]. Second, the handheld US images were obtained straight from the Butterfly network cloud. In contrast, the traditional US photographs were imported into the PC after being retrieved from the machine’s hard drive. The latter could have led to picture deterioration during the transfer.

Although handheld ultrasonography technology can show good sensitivity in detecting retinal detachment in some cases, usage in an ophthalmology emergency department should be used cautiously. It can be limiting and may be contraindicated, especially when there is suspicion of intraocular hemorrhage or globe rupture. These situations necessitate more thorough imaging and meticulous medical evaluation to prevent the worsening of the injury. The handheld device may have limitations in identifying subtle indications of these severe diseases, and its usage may be inappropriate in circumstances requiring prompt surgical intervention. Thus, it is advisable to maintain traditional imaging techniques as the prevailing approach in such crucial situations until additional validation studies can substantiate the broader use of portable devices.

## 5. Conclusions

In this study, we showed that a handheld ultrasound (Butterfly iQ+) device accurately diagnoses retinal detachment in an emergency setting. Butterfly iQ+ could be a valuable diagnostic tool in ED ocular diagnosis due to its accessibility and portability. It allows the emergency doctor or general practitioner to use its telemedicine functions to consult the eye specialist in a remote setting. However, significant improvements in vitreous body resolution and better maneuverability will be needed for widespread clinical application. It should also be noted that eye ultrasound requires appropriate training and experience, making it essential to use in a telemedicine approach or by trained professionals.

## Figures and Tables

**Table 1:** Performance for each instrument per diagnosis.

	Accutome B-Scan Pro		Butterfly iQ+	
	Sensitivity	Specificity	Sensitivity	Specificity
RD	94.4	91.2	91.7	95.6
VH	66.7	86.8	66.7	97.7 *
PVD	48.5	87.2	27.2 *	88.0
Pathologic vs. Healthy	97.8	70.0	73.3 *	80.0
*Accuracy*	*70.7% (62.7–77.8%)*		***69.3% (61.3–76.6%)***	

* Butterfly iQ + is significantly different from Accutome B-scan Pro.

**Table 2:** Concordance between the four raters on the four diagnoses for each ultrasound probe and between ultrasound probes.

	Accutome B-Scan Pro		Butterfly iQ+		Between Instruments	
	Concordance	*p*-Value	Concordance	*p*-Value	Concordance	*p*-Value
RD	0.775	<0.001	0.789	<0.001	0.698	<0.001
VH	0.512	<0.001	0.668	<0.001	0.355	<0.001
PVD	0.227	0.005	−0.078	0.337	0.051	0.528
Healthy	0.678	<0.001	0.653	<0.001	0.269	0.001
Total	0.569	<0.001	0.552	<0.001	0.359	<0.001

**Table 3:** Quality of image rating distribution according to probes and diagnosis.

Rating	RD		VH		PVD		Healthy	
	Accutome	iQ+	Accutome	iQ+	Accutome	iQ+	Accutome	iQ+
1	0 (0.0%)	0 (0.0%)	0 (0.0%)	0 (0.0%)	0 (0.0%)	0 (0.0%)	0 (0.0%)	0 (0.0%)
2	0 (0.0%)	2 (5.6%)	2 (9.5%)	3 (14.3%)	2 (6.1%)	3 (9.1%)	5 (8.3%)	3 (5.0%)
3	8 (22.2%)	9 (25.0%)	6 (28.6%)	4 (19.0%)	11 (33.3%)	17 (51.5%)	26 (43.3%)	19 (31.7%)
4	18 (50.0%)	22 (61.1%)	10 (47.6%)	14 (66.7%)	18 (54.5%)	13 (39.4%)	16 (26.7%)	34 (56.7%)
5	10 (27.8%)	3 (8.3%)	3 (14.3%)	0 (0.0%)	2 (6.1%)	0 (0.0%)	13 (21.7%)	4(6.7%)

**Table 4:** Confidence of image rating distribution according to probes and diagnosis.

Rating	RD		VH		PVD		Healthy	
	Accutome	iQ+	Accutome	iQ+	Accutome	iQ+	Accutome	iQ+
1	0 (0.0%)	0 (0.0%)	0 (0.0%)	0 (0.0%)	0 (0.0%)	0 (0.0%)	0 (0.0%)	0 (0.0%)
2	0 (0.0%)	2 (5.6%)	3 (14.3%)	3 (14.3%)	6 (18.2%)	5 (15.2%)	5 (8.3%)	13 (21.7%)
3	5 (13.9%)	16 (44.4%)	8 (38.1%)	8 (38.1%)	6 (18.2%)	12 (36.4%)	18 (30.0%)	20 (33.3%)
4	10 (27.8%)	7 (19.4%)	5 (23.8%)	8 (38.1%)	14 (42.4%)	8 (24.2%)	20 (33.3%)	11 (18.3%)
5	21 (58.3%)	11 (30.6%)	5 (23.8%)	2 (9.5%)	7 (21.2%)	8 (24.2%)	17 (28.3%)	16 (26.7%)

## Data Availability

Data are available upon request.
